# Bis(trimethyl­phenyl­ammonium) μ-oxalato-bis­[oxidodiperoxido­molybdate(VI)]

**DOI:** 10.1107/S1600536812045850

**Published:** 2012-11-10

**Authors:** Ayaka Oba, Masato Hashimoto

**Affiliations:** a Department of Material Science and Chemistry, Faculty of Systems Engineering, Wakayama University, Sakaedani 930, Wakayama 640-8510, Japan

## Abstract

A trimethyl­phenyl­ammonium salt of a dinuclear μ-oxalate complex of diperoxidomonomolybdate units, (C_9_H_14_N)_2_[Mo_2_(C_2_O_4_)(O_2_)_4_O_2_], was obtained from an acidic aqueous solution; the dianion is located about a centre of inversion. Each Mo atom bears two peroxide groups together with one O atom from the oxalate group in its equatorial positions and one terminal O atom as well as another O atom from the oxalate in axial positions. The oxalate group acts as a tetra­dentate bridging ligand and bridges between the diperoxidomolybdate units.

## Related literature

For the structure of the closely related tetra­butyl­ammonium peroxidotungstate analogue, see Hashimoto *et al.* (1987 ▶). For the structures of related molybdate complexes, see Stomberg & Olson (1985 ▶); Bayot *et al.* (2004 ▶).
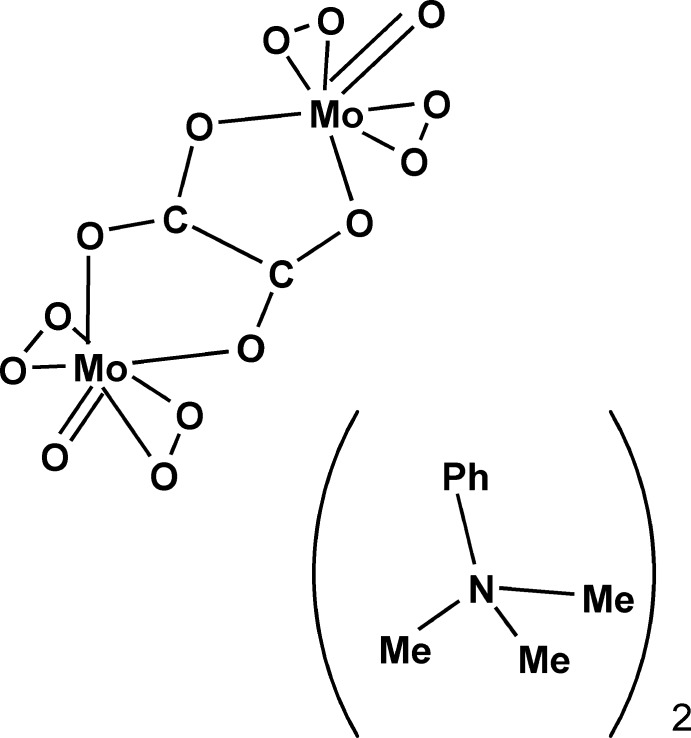


## Experimental

### 

#### Crystal data

(C_9_H_14_N)_2_[Mo_2_(C_2_O_4_)(O_2_)_4_O_2_]
*M* *_r_* = 712.32
Monoclinic, 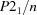

*a* = 9.860 (2) Å
*b* = 9.975 (2) Å
*c* = 13.691 (3) Å
β = 94.023 (3)°
*V* = 1343.2 (5) Å^3^
*Z* = 2
Mo *K*α radiation
μ = 1.00 mm^−1^
*T* = 93 K
0.20 × 0.17 × 0.17 mm

#### Data collection

Rigaku Saturn724+ diffractometer
Absorption correction: numerical (*NUMABS*; Higashi, 2000 ▶) *T*_min_ = 0.888, *T*_max_ = 0.914
11791 measured reflections
3843 independent reflections
3621 reflections with *I* > 2σ(*I*)
*R*_int_ = 0.021

#### Refinement

*R*[*F* ^2^ > 2σ(*F* ^2^)] = 0.029
*wR*(*F* ^2^) = 0.080
*S* = 1.06
3843 reflections
172 parameters
H-atom parameters not refined
Δρ_max_ = 1.84 e Å^−3^
Δρ_min_ = −0.69 e Å^−3^

### 

Data collection: *CrystalClear SM* (Rigaku, 2008 ▶); cell refinement: *CrystalClear SM*; data reduction: *CrystalClear SM*; program(s) used to solve structure: *SHELXS97* (Sheldrick, 2008 ▶); program(s) used to refine structure: *SHELXL97* (Sheldrick, 2008 ▶); molecular graphics: *ORTEP-3 for Windows* (Farrugia, 1997 ▶); software used to prepare material for publication: *SHELXL97*.

## Supplementary Material

Crystal structure: contains datablock(s) I, global. DOI: 10.1107/S1600536812045850/im2410sup1.cif

Structure factors: contains datablock(s) I. DOI: 10.1107/S1600536812045850/im2410Isup2.hkl

Additional supplementary materials:  crystallographic information; 3D view; checkCIF report

## Figures and Tables

**Fig. 1.**
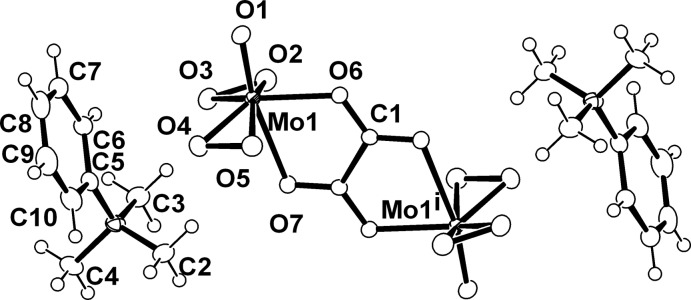
Molecular structure of the title compound with thermal ellipsoinds drawn at the 50% probability level for non-H atoms. Symmetry code: (i) 1 - x, 1 - y, -z.

## supplementary crystallographic information

### Comment

A novel dinuclear peroxidomolybdate complex with a bridging oxalate ligand was crystallized as a trimethylphenylammonium salt in the course of investigating complex formation of oxalate and peroxomolybdate in aiming at preparing an inorganic-organic hybrid material.

The compound consists of two trimethylphenylammonium cations and one dinuclear oxalato complex of diperoxidomolybdate (Figure 1). In the complex anion two diperoxidomolybdates are bridged by a tetradentate µ^2^ chelating oxalato ligand. Each molybdenum atom has two peroxo groups in its equatorial positions. The fifth equatorial position and one of the axial positions are occupied by O atoms of the oxalate ligand. The second axial position is occupied by the terminal oxo ligand. Each molybdenum atom thus exhibits a distorted pentagonal bipyramidal geometry, which is common in peroxidomolybdate compounds. C1, C1^i^, O6, O6^i^, O7, O7^i^, Mo1, Mo1^i^, O1 and O1^i^ (symmetry operation, ^i^(1 - *x*, 1 - *y*, -*z*)) are coplanar with a maximum deviation of 0.0240 (11) Å (O6) from the corresponding least square plane. Oxygen atoms of both peroxo ligands at each Mo atom are also coplanar. The deviation of the Mo atom from the least square plane toward the terminal oxygen atom is -0.401 (1) Å. These planes are almost perpendicular to the initially mentioned least square plane with dihedral angles 89.86 (5)°. There is no abnormal bond length and bond angles in the anion. Among Mo—O bonds in the equatorial plane Mo1—O6 (2.092 (1) Å) shows a slightly longer distance than others. C1—O7 (1.232 (2) Å) is slightly shorter than C1—O6 (1.275 (2) Å) probably because of weaker interaction of Mo1—O7 than Mo1—O6. Bond lengths in the trimethylphenylammonium cations are also normal. No special intermolecular interaction is observed in the packing.

The tungstate analogue of the present anion, [W_2_(C_2_O_4_)(O_2_)_4_O_2_], was reported as a tetrabutylanmonium salt by one of the authors (Hashimoto *et al.*, 1987). The geometry of the complex anion of the tungsten compound is essentially identical to the molybdate one. The potassium salt of a mononuclear oxalato complex of a peroxidomolybdate was reported by Stomberg (Stomberg *et al.*, 1985). The oxalate group of the mononuclear complex chelates to a molybdenum atom to form a Mo—O—C—C—O five-membered ring as found in the title compound. C—O distances of the non-coordinated O atoms of the oxalate group in the mononuclear complex (1.21 (1) and 1.22 (1) Å) are considerably shorter than C—O distances towards coordinationg oxygen atoms (1.30 (1) and 1.28 (1) Å), reflecting the coordination to the metal center. On the contrary, the 2:2 complex reported by Bayot *et al.*, shows a different structural feature. The complex anion can be regarded as a dimer of Stomberg's mononuclear complex. However, one of the peroxo groups is replaced by two hydroxo groups to form a dimer of oxalatomonoperoxidomolybdate moieties doubly bridged by two µ^2^-OH groups (Bayot *et al.*, 2004). The hydroxo groups thus occupy equatorial positions of the molybdenum atom. Distances and angles in the 2:2 complex show similar tendencies as Stomberg's 1:1 and the present 2:1 (Mo:oxalate) complexes, such as longer Mo—O(oxalate) distance than other Mo—O on the equatorial plane, and shorter C—O(equatorial) distances compared to C—O(axial) bonds.

### Experimental

The single-crystal subjected to the X-ray analysis was obtained in the following way. Sodium molybdate dihydrate (14.52 g, 0.0600 mol) and oxalic acid dihydrate (3.78 g, 0.0300 mol) were dissolved in *ca* 60 ml water. To this solution 10 ml of 30% H_2_O_2_ (*ca* 0.10 mol) was added and the volume was adjusted to 100 ml. The pH of the resulted solution was adjusted to 2 with *ca* 13 mol *L*^-1^ nitric acid. To the solution was added 1.0 ml of 1.0 mol *L*^-1^ aqueous trimethylphenylammonium chloride solution and the mixture was kept at room temperature. Block shaped crystals appeared in one day (yield 25%).

### Refinement

Hydrogen atoms have been calculated in idealized positions with C–H bond lengths of 0.93 Å (aromatic) and 0.96 Å (methyl). They were refined using a riding model with U_eq_(H) = 1.2 × U_iso_(C) for aromatic and U_eq_(H) = 1.5 × U_iso_(C) for methyl groups.

### Crystal data

**Table:**

(C_9_H_14_N)_2_[Mo_2_(C_2_O_4_)(O_2_)_4_O_2_]	*F*(000) = 716
*M**_r_* = 712.32	*D*_x_ = 1.761 Mg m^−^^3^
Monoclinic, *P*2_1_/*n*	Mo *K*α radiation, λ = 0.71069 Å
Hall symbol: -P 2yn	Cell parameters from 5064 reflections
*a* = 9.860 (2) Å	θ = 2.9–30.0°
*b* = 9.975 (2) Å	µ = 1.00 mm^−^^1^
*c* = 13.691 (3) Å	*T* = 93 K
β = 94.023 (3)°	Block, pale yellow
*V* = 1343.2 (5) Å^3^	0.20 × 0.17 × 0.17 mm
*Z* = 2	

### Data collection

**Table:**

Rigaku Saturn724+ diffractometer	3843 independent reflections
Radiation source: fine-focus rotating anode	3621 reflections with *I* > 2σ(*I*)
Graphite monochromator	*R*_int_ = 0.021
Detector resolution: 28.5174 pixels mm^-1^	θ_max_ = 30.0°, θ_min_ = 2.5°
CCD scans	*h* = −13→13
Absorption correction: numerical (*NUMABS*; Higashi, 2000)	*k* = −14→10
*T*_min_ = 0.888, *T*_max_ = 0.914	*l* = −19→16
11791 measured reflections	

### Refinement

**Table:**

Refinement on *F*^2^	Primary atom site location: Patterson
Least-squares matrix: full	Secondary atom site location: difference Fourier map
*R*[*F*^2^ > 2σ(*F*^2^)] = 0.029	Hydrogen site location: inferred from neighbouring sites
*wR*(*F*^2^) = 0.080	H-atom parameters not refined
*S* = 1.06	*w* = 1/[σ^2^(*F*_o_^2^) + (0.0409*P*)^2^ + 1.5201*P*] where *P* = (*F*_o_^2^ + 2*F*_c_^2^)/3
3843 reflections	(Δ/σ)_max_ = 0.001
172 parameters	Δρ_max_ = 1.84 e Å^−^^3^
0 restraints	Δρ_min_ = −0.69 e Å^−^^3^

### Special details

**Table:**

Geometry. All e.s.d.'s (except the e.s.d. in the dihedral angle between two l.s. planes) are estimated using the full covariance matrix. The cell e.s.d.'s are taken into account individually in the estimation of e.s.d.'s in distances, angles and torsion angles; correlations between e.s.d.'s in cell parameters are only used when they are defined by crystal symmetry. An approximate (isotropic) treatment of cell e.s.d.'s is used for estimating e.s.d.'s involving l.s. planes.
Refinement. Refinement of *F*^2^ against ALL reflections. The weighted *R*-factor *wR* and goodness of fit *S* are based on *F*^2^, conventional *R*-factors *R* are based on *F*, with *F* set to zero for negative *F*^2^. The threshold expression of *F*^2^ > σ(*F*^2^) is used only for calculating *R*-factors(gt) *etc*. and is not relevant to the choice of reflections for refinement. *R*-factors based on *F*^2^ are statistically about twice as large as those based on *F*, and *R*- factors based on ALL data will be even larger.

### Fractional atomic coordinates and isotropic or equivalent isotropic displacement parameters (Å^2^)

**Table:**

	*x*	*y*	*z*	*U*_iso_*/*U*_eq_	
Mo1	0.354779 (14)	0.254491 (13)	0.035607 (10)	0.01536 (7)	
O1	0.38434 (13)	0.09445 (13)	0.00536 (9)	0.0205 (2)	
O2	0.45582 (16)	0.27065 (15)	0.16277 (11)	0.0248 (3)	
O3	0.30966 (17)	0.24598 (13)	0.17011 (11)	0.0249 (3)	
O4	0.16612 (15)	0.28161 (16)	−0.00396 (12)	0.0272 (3)	
O5	0.25414 (14)	0.31884 (15)	−0.08269 (10)	0.0269 (3)	
O6	0.52797 (13)	0.32966 (12)	−0.02533 (9)	0.0192 (2)	
O7	0.35239 (13)	0.48958 (13)	0.06131 (11)	0.0231 (3)	
C1	0.55099 (17)	0.45548 (17)	−0.02518 (12)	0.0172 (3)	
N1	−0.00978 (16)	0.42328 (15)	0.22184 (11)	0.0198 (3)	
C2	0.0346 (2)	0.5196 (2)	0.14525 (14)	0.0258 (4)	
H2A	0.0534	0.6055	0.1747	0.039*	
H2B	−0.0366	0.5285	0.0941	0.039*	
H2C	0.1151	0.4859	0.1182	0.039*	
C3	0.1020 (2)	0.4182 (2)	0.30216 (14)	0.0248 (4)	
H3A	0.1181	0.5067	0.3281	0.037*	
H3B	0.1836	0.3848	0.2764	0.037*	
H3C	0.0758	0.3599	0.3533	0.037*	
C4	−0.1357 (2)	0.4769 (2)	0.26505 (14)	0.0277 (4)	
H4A	−0.1165	0.5635	0.2935	0.042*	
H4B	−0.1629	0.4166	0.3147	0.042*	
H4C	−0.2077	0.4849	0.2144	0.042*	
C5	−0.04052 (17)	0.28802 (19)	0.17753 (12)	0.0187 (3)	
C6	0.02069 (18)	0.17351 (18)	0.21818 (13)	0.0213 (3)	
H6	0.0821	0.1795	0.2728	0.026*	
C7	−0.0117 (2)	0.0493 (2)	0.17563 (16)	0.0274 (4)	
H7	0.0286	−0.0281	0.2021	0.033*	
C8	−0.1029 (2)	0.0402 (2)	0.09455 (17)	0.0321 (4)	
H8	−0.1228	−0.0427	0.0659	0.039*	
C9	−0.1647 (2)	0.1554 (3)	0.05603 (16)	0.0339 (5)	
H9	−0.2273	0.1492	0.0022	0.041*	
C10	−0.1338 (2)	0.2797 (2)	0.09723 (15)	0.0275 (4)	
H10	−0.1754	0.3567	0.0712	0.033*	

### Atomic displacement parameters (Å^2^)

**Table:**

	*U*^11^	*U*^22^	*U*^33^	*U*^12^	*U*^13^	*U*^23^
Mo1	0.01690 (9)	0.01264 (9)	0.01686 (9)	−0.00015 (4)	0.00347 (6)	0.00104 (4)
O1	0.0238 (6)	0.0166 (6)	0.0214 (6)	−0.0016 (5)	0.0033 (5)	−0.0005 (4)
O2	0.0280 (7)	0.0265 (6)	0.0195 (6)	−0.0020 (5)	−0.0003 (5)	−0.0021 (5)
O3	0.0313 (8)	0.0228 (7)	0.0218 (7)	−0.0015 (5)	0.0106 (6)	−0.0004 (4)
O4	0.0194 (6)	0.0273 (7)	0.0351 (8)	0.0008 (5)	0.0046 (5)	0.0042 (6)
O5	0.0250 (6)	0.0293 (7)	0.0264 (7)	0.0021 (5)	0.0007 (5)	0.0080 (6)
O6	0.0195 (6)	0.0141 (5)	0.0250 (6)	0.0007 (4)	0.0079 (5)	0.0004 (4)
O7	0.0218 (6)	0.0161 (6)	0.0328 (7)	0.0004 (5)	0.0122 (5)	0.0001 (5)
C1	0.0171 (7)	0.0152 (7)	0.0198 (7)	0.0021 (5)	0.0047 (6)	0.0010 (6)
N1	0.0242 (7)	0.0187 (7)	0.0170 (6)	0.0050 (5)	0.0059 (5)	0.0003 (5)
C2	0.0348 (10)	0.0207 (8)	0.0232 (8)	0.0046 (7)	0.0110 (7)	0.0048 (7)
C3	0.0314 (9)	0.0223 (9)	0.0202 (8)	0.0007 (7)	−0.0008 (7)	−0.0044 (7)
C4	0.0319 (9)	0.0300 (10)	0.0227 (8)	0.0127 (8)	0.0127 (7)	0.0034 (7)
C5	0.0175 (7)	0.0223 (8)	0.0168 (7)	0.0007 (6)	0.0043 (6)	−0.0017 (6)
C6	0.0206 (7)	0.0206 (8)	0.0227 (8)	0.0007 (6)	0.0024 (6)	−0.0010 (6)
C7	0.0248 (8)	0.0215 (9)	0.0368 (10)	−0.0017 (7)	0.0081 (7)	−0.0035 (8)
C8	0.0258 (9)	0.0358 (11)	0.0359 (11)	−0.0122 (8)	0.0092 (8)	−0.0141 (9)
C9	0.0259 (9)	0.0486 (13)	0.0267 (9)	−0.0091 (9)	−0.0020 (7)	−0.0075 (9)
C10	0.0215 (9)	0.0393 (10)	0.0214 (8)	0.0023 (8)	−0.0007 (7)	0.0020 (8)

### Geometric parameters (Å, º)

**Table:**

Mo1—O1	1.6799 (13)	C2—H2C	0.9600
Mo1—O4	1.9198 (15)	C3—H3A	0.9600
Mo1—O3	1.9264 (16)	C3—H3B	0.9600
Mo1—O5	1.9479 (14)	C3—H3C	0.9600
Mo1—O2	1.9510 (15)	C4—H4A	0.9600
Mo1—O6	2.0915 (12)	C4—H4B	0.9600
Mo1—O7	2.3716 (14)	C4—H4C	0.9600
O2—O3	1.472 (2)	C5—C10	1.386 (3)
O4—O5	1.478 (2)	C5—C6	1.390 (3)
O6—C1	1.275 (2)	C6—C7	1.397 (3)
O7—C1^i^	1.232 (2)	C6—H6	0.9300
C1—O7^i^	1.232 (2)	C7—C8	1.382 (3)
C1—C1^i^	1.540 (3)	C7—H7	0.9300
N1—C5	1.501 (2)	C8—C9	1.387 (4)
N1—C3	1.502 (2)	C8—H8	0.9300
N1—C2	1.509 (2)	C9—C10	1.387 (3)
N1—C4	1.511 (2)	C9—H9	0.9300
C2—H2A	0.9600	C10—H10	0.9300
C2—H2B	0.9600		
			
O1—Mo1—O4	104.23 (7)	N1—C2—H2A	109.5
O1—Mo1—O3	104.45 (6)	N1—C2—H2B	109.5
O4—Mo1—O3	89.53 (7)	H2A—C2—H2B	109.5
O1—Mo1—O5	101.26 (6)	N1—C2—H2C	109.5
O4—Mo1—O5	44.92 (6)	H2A—C2—H2C	109.5
O3—Mo1—O5	132.14 (7)	H2B—C2—H2C	109.5
O1—Mo1—O2	102.24 (6)	N1—C3—H3A	109.5
O4—Mo1—O2	131.62 (7)	N1—C3—H3B	109.5
O3—Mo1—O2	44.63 (7)	H3A—C3—H3B	109.5
O5—Mo1—O2	155.95 (6)	N1—C3—H3C	109.5
O1—Mo1—O6	94.69 (6)	H3A—C3—H3C	109.5
O4—Mo1—O6	129.72 (6)	H3B—C3—H3C	109.5
O3—Mo1—O6	130.31 (6)	N1—C4—H4A	109.5
O5—Mo1—O6	86.14 (6)	N1—C4—H4B	109.5
O2—Mo1—O6	86.92 (6)	H4A—C4—H4B	109.5
O1—Mo1—O7	168.51 (5)	N1—C4—H4C	109.5
O4—Mo1—O7	83.28 (6)	H4A—C4—H4C	109.5
O3—Mo1—O7	84.05 (5)	H4B—C4—H4C	109.5
O5—Mo1—O7	77.75 (6)	C10—C5—C6	120.92 (19)
O2—Mo1—O7	78.21 (6)	C10—C5—N1	118.58 (17)
O6—Mo1—O7	73.84 (4)	C6—C5—N1	120.47 (15)
O3—O2—Mo1	66.80 (9)	C5—C6—C7	118.82 (18)
O2—O3—Mo1	68.57 (8)	C5—C6—H6	120.6
O5—O4—Mo1	68.55 (8)	C7—C6—H6	120.6
O4—O5—Mo1	66.53 (8)	C8—C7—C6	120.7 (2)
C1—O6—Mo1	120.15 (10)	C8—C7—H7	119.7
C1^i^—O7—Mo1	111.35 (11)	C6—C7—H7	119.7
O7^i^—C1—O6	125.41 (15)	C7—C8—C9	119.67 (19)
O7^i^—C1—C1^i^	118.07 (19)	C7—C8—H8	120.2
O6—C1—C1^i^	116.52 (18)	C9—C8—H8	120.2
C5—N1—C3	112.48 (14)	C8—C9—C10	120.51 (19)
C5—N1—C2	110.57 (14)	C8—C9—H9	119.7
C3—N1—C2	107.24 (15)	C10—C9—H9	119.7
C5—N1—C4	109.18 (15)	C5—C10—C9	119.4 (2)
C3—N1—C4	107.85 (14)	C5—C10—H10	120.3
C2—N1—C4	109.44 (14)	C9—C10—H10	120.3

Symmetry code: (i) −*x*+1, −*y*+1, −*z*.
